# Zero prevalence of extended spectrum beta-lactamase-producing bacteria in 300 breeding Collared Flycatchers in Sweden

**DOI:** 10.3402/iee.v3i0.20909

**Published:** 2013-07-25

**Authors:** Josef D. Järhult, Johan Stedt, Lars Gustafsson

**Affiliations:** 1 Section for Infectious Diseases, Department of Medical Sciences, Uppsala University, Uppsala, Sweden; 2 Section for Zoonotic Ecology and Epidemiology, School of Natural Sciences, Linnaeus University, Kalmar, Sweden; 3 Section for Animal Ecology, Department of Ecology and Genetics, Uppsala University, Uppsala, Sweden

**Keywords:** ESBL, E. coli, Passerines, wild birds, antibiotic resistance, surveillance

## Abstract

Wild birds are important indicators and potential spreaders of antibiotic resistance. The order *Passerines* is scarcely studied apart from *Corvus* sp. but extended spectrum beta-lactamases (ESBLs) has been found in Blackbirds. We tested 300 fecal samples from a well-studied population of Collared Flycatchers (*Ficedula albicollis*) at the Island of Gotland in Sweden and found no ESBL-producing bacteria. These results support the idea of ‘ecological guild’ as Blackbirds are ground-foraging invertebrate feeders, whereas Collared Flycatchers are aerial insectivores not regularly coming into contact with fecal contaminations and therefore less prone to acquire pathogens spread by the fecal–oral route.

Antibiotic-resistant bacteria are a major problem for the human health care and the difficulties are rapidly increasing. Most studies so far have focused on human and veterinary medicine, but there is a growing research interest also in the environmental resistance situation. Resistance genes have been found in isolated locations such as the water in Antarctica (1), indicating that the problem has spread also to the most remote parts of our environment. Birds are of interest in this sense both as indicators for the prevalence of resistance in the environment and as a potential mechanism for spread through migration.

Several studies have focused on the prevalence of general resistance in *E. coli* and extended spectrum beta-lactamases (ESBLs) in the gut flora of different bird species, especially gulls (*Larus* sp.). Gulls often live close to humans and feed on our waste products, and some studies on gulls have shown that they regularly carry a high prevalence of general resistance in *E. coli* and ESBL-producing strains (2–5). Other birds living in proximity to humans or feeding on human waste products have also been shown to carry resistant bacteria, for example, Feral Pigeons (*Columba livia*) (6) and Red-Billed Choughs (*Pyrrhocorax pyrrhocorax*) (7). Bird groups without close contact to humans have also been shown to carry resistant bacteria, foremost waterfowl and birds of prey, such as Canada Geese (*Branta canadensis*) (8, 9), Mallards (*Anas platyrhynchos*) (10) and different species of raptors (*Accipitridae* *sp*) (11–13). Here, contaminated water or prey is most likely the source of the resistant bacteria. It has been demonstrated that there is a concordance between human-associated *E. coli* strains and the resistant *E. coli* strains found in gulls, indicating that the resistant bacteria isolated from birds is a result of human dissemination (4, 5).

The order *Passerines* that includes more than half of all bird species are very poorly studied except for *Corvus* sp. (7). To our knowledge, only two studies analyzing samples collected from *Passerines* other than *Corvus* sp. have been published. One study included 154 Song Thrushes (*Turdus philomelos*) in Portugal and no ESBL-producing bacteria were found (14), and in one study from Germany, samples from 99 *Passerines* were analyzed and ESBLs of the genotype CTX-M 15 were found in two Blackbirds (*Turdus merula*) (15). Furthermore, it has been shown that White-throated Dippers (*Cinclus cinclus*) and Garden Warblers (*Sylvia borin*) can carry *E. coli* resistant to several antibiotics, but no ESBL was found (16).

Increasingly, ecologists and evolutionary biologists are realizing the value of long-term data on individually marked organisms followed in long-term studies. Since 1980, Collared Flycatchers (*Ficedula albicollis*) have been studied at the Gotland Island in Sweden with regard to life history evolution, sexual selection, and immune-ecology. Especially, we have studied how these aspects are linked together, both at the phenotypic and the underlying genetic variation level (17–21). Collared Flycatchers breed in Eastern Europe have their northern breeding border in southeastern Sweden and during winter they migrate to East Africa. Flycatchers often breed close to humans and feed on small invertebrates.

We wanted to examine the prevalence of ESBL-producing bacteria in the well-defined and well-studied population of Collared Flycatchers at the Island of Gotland, thereby extending the knowledge of antibiotic resistance among passerines. Therefore, we collected fecal samples from 300 Collared Flycatchers in the population during the summer of 2011.

All 300 fecal samples were pooled five by five and screened for ESBL-producing *Bacteria* using earlier described methods (3). In brief, all samples were first enriched in brain heart infusion broth (Becton Dickinson, Franklin Lakes, NJ, USA), supplemented with 16 mg/L vancomycin, for 18–24 h at 37°C. Each sample was subsequently inoculated on a chromIDTM ESBL plate (bioMérieux, Marcy l'Etoile, France), according to the manufacturer's instructions. On the chromIDTM ESBL plates, no growth of Gram-negative bacteria was recognized despite bacterial growth in all of the broths. Some of the pooled samples were also spread on blood agar plates to confirm bacterial growth.

Our results from Collared Flycatchers with an absence of ESBL-producing bacteria are in concordance with the study on Song Thrushes in Portugal (14). In the German study, however, ESBLs were found in two Blackbirds, demonstrating prevalence among *Passerines* (15). A drawback of the German study is that the numbers of birds in relation to species and feeding habits were not reported. Feeding habits – or ‘ecological guild’ – have been shown to be closely correlated to the prevalence of *Campylobacter* sp. (22). Both campylobacter and ESBL-producing bacteria are transmitted via the fecal–oral route and therefore the same pattern of prevalence seems likely for the ESBLs. Blackbirds – where ESBLs were found – are ground-foraging invertebrate feeders more likely to come in contact with feces-contaminated material. Collared Flycatchers on the other hand, although belonging to the same order, are aerial insectivores not regularly coming into contact with fecal contaminations. However, insectivores such as White-throated Dippers and Garden Warblers have been found to carry resistant *E. coli*, even if only from one bird of each species and not ESBL-producing strains (16). White-throated Dippers collect insects from water and thus might come into contact with aquatic contaminations. Garden Warblers are not strictly aerial insectivores, but also eat fruits and berries before migration; this feeding behavior may increase the risk of transmission of resistant bacteria. Our study supports the idea that ecological guild is an important factor to determine the prevalence of pathogens that are transmitted through the fecal–oral route and that strictly aerial insectivores are unlikely to be colonized with ESBL-producing bacteria.
